# IL-19 Up-Regulates Mucin 5AC Production in Patients With Chronic Rhinosinusitis *via* STAT3 Pathway

**DOI:** 10.3389/fimmu.2019.01682

**Published:** 2019-07-17

**Authors:** Xiaoping Lai, Xia Li, Lihong Chang, Xiaohong Chen, Zizhen Huang, Hongwei Bao, Jiancong Huang, Luoying Yang, Xifu Wu, Zhiyuan Wang, Joseph A. Bellanti, Song Guo Zheng, Gehua Zhang

**Affiliations:** ^1^Department of Otorhinolaryngology, The Third Affiliated Hospital of Sun Yat-sen University, Guangzhou, China; ^2^Department of Pediatrics and Microbiology-Immunology, Georgetown University Medical Center, Washington, DC, United States; ^3^Department of Internal Medicine, Ohio State University College of Medicine, Columbus, OH, United States

**Keywords:** IL-19, MUC5AC, chronic rhinosinusitis, STAT3, nasal epithelium cells

## Abstract

The mucin gene, MUC5AC, is highly expressed both in chronic respiratory inflammatory diseases and inflammatory bowel disease where mucin secretion is regulated by members of the interleukin IL-20 subfamily. This study was conducted to determine the roles and mechanisms of IL-19, a member of the IL-20 subfamily, in regulating MUC5AC production in chronic rhinosinusitis (CRS). We analyzed the expression of mucin and MUC5AC in the nasal mucosa of patients with CRS through periodic acid Schiff (PAS) staining and immunohistochemical examination. Real-time quantitative PCR, ELISA, confocal microscopy and western blotting were used to measure MUC5AC expression in primary human nasal epithelium cells (PHNECs) stimulated with recombinant human IL-19 (rhIL-19), IL-19 receptor siRNA transfection or a control. The involvement of the STAT3 signaling pathway was examined using cryptotanshinone (CRY, an inhibitor of STAT3). Mucin and MUC5AC were significantly increased in mucosa of CRS patients with/without nasal polyps compared to mucosa isolated from controls who had no CRS, but there were no significant differences between these two groups. Pretreatment with rhIL-19 up-regulated the expression of MUC5AC levels in PHNECs. Knockdown of IL-20R2 and pretreatment with CRY attenuated MUC5AC production induced by rhIL-19. We propose that IL-19 up-regulates MUC5AC-induced mucin production *via* the STAT3 pathway in CRS, highlighting the important role IL-19 may play in mucin production in chronic respiratory diseases.

## Introduction

Mucus over-production is an important pathophysiological manifestation of chronic obstructive pulmonary disease (COPD), asthma, bronchiectasis, cystic fibrosis, and other chronic airway inflammatory diseases (1). Mucus is mainly produced by goblet cells following stimulation by a wide range of stimuli that have been shown to cause up-regulation in the number of mucus-producing goblet cells (2). The major macromolecular constituents of mucus are mucins, proteins encoded by mucin (MUC) genes (3). To date, more than 20 mucin genes have been identified (4). Among the proteins encoded by these genes, MUC5AC is the predominant mucin expressed in goblet cells in human airways (5, 6).

Chronic rhinosinusitis (CRS) is a heterogeneous disorder of the paranasal sinuses characterized by local inflammation which persists for more than 12 weeks. According to the European Position Paper on Rhinosinusitis and Nasal Polyps 2012 (EP^3^OS2012), CRS can be divided into two groups, chronic rhinosinusitis with nasal polyps (CRSwNP) and chronic rhinosinusitis without nasal polyps (CRSsNP) (7). One of the remarkable features of CRS is mucus over-production, which manifests as nasal discharge. It has been suggested that MUC5AC was significantly increased in epithelial goblet cells in CRS (8, 9). Although many studies showed that MUC5AC was closely related to airway inflammation and goblet cells metaplasia, the molecular mechanism of MUC5AC production in CRS remains unclear.

A recent study by Andhou et al. (10) suggests that IL-24, a member of the IL-20 subfamily, not only participates in tissue remodeling but also plays an important role in mucin deposition in the epithelial cells in inflammatory bowel disease (10). We also found that IL-19, another member of the IL-20 subfamily, and its receptors, IL-20R1 and IL-20R2, were highly expressed in CRSwNP (Supplementary Figure 3). However, whether IL-19 is involved in mucus production in CRS and its mechanism remains unknown.

The aim of this study is to investigate the expressions of mucin and MUC5AC in CRS mucosa and to determine whether and how IL-19 regulates MUC5AC production in CRS.

## Materials and Methods

### Study Subjects

The study was approved by the Institutional Review Board of the Third Affiliated Hospital of Sun Yat-sen University (No. [2016]2-26). All enrolled subjects provided prior written informed consent. Human nasal tissues were obtained during endoscopic sinus surgery (ESS) from 40 patients with CRS (30 CRSwNP and 10 CRSsNP) diagnosed according to EP3OS2012 and 15 control subjects undergoing ESS without CRS, in the period between August 2016 to April 2017. More detailed descriptions of patient characteristics are shown in Table 1. Nasal polyps and ostiomeatal complex (OMC) mucosal tissues were obtained from the CRS patients and inferior turbinate mucosal tissues were obtained from the control subjects. Exclusion criteria included patients with autoimmune diseases, aspirin intolerance triad, primary ciliary dyskinesia and cystic fibrosis as well as patients who had received antibiotics or glucocorticoids for a period of at least 1 month prior to surgery.

**Table 1:** Patient characteristics.

	**Control**	**CRSsNP**	**CRSwNP**
Total subject number	15	10	30
Gender, male/female	12/3	7/3	15/15
Age (y), mean ± *SD*	35.9 ± 14.4	35.8 ± 14.9	36.9 ± 12.3
Atopy, n (%)	3 (20.0%)	2 (20.0%)	8 (26.7%)
Asthma, n (%)	0	0	2 (6.7%)
Smoking, n (%)	1 (6.7%)	1 (10.0%)	3 (10.0%)

Nasal polyps obtained from patients in the CRSwNP group were stored in PBS supplemented by penicillin, streptomycin and amphotericin B and prepared for cell culture. OMC mucosal tissues from the CRSwNP and CRSsNP groups as well as the inferior turbinate mucosal specimens from the control subjects were immediately fresh frozen in liquid nitrogen and stored at −80°C until analyzed.

### Periodic Acid Schiff (PAS) Staining

All tissues were fixed with 4% paraformaldehyde, embedded in paraffin, cut into 4 μm thick sections and then stained with PAS following routine staining procedures. The quantity of mucin production was assessed by measuring the percentage of PAS-positive cells per total epithelial cells. Each section had five randomly selected regions evaluated. The average PAS-positive percentage was calculated for each section.

### Immunohistochemistry (IHC) Staining for MUC5AC

All tissues were fixed with 4% paraformaldehyde, embedded in paraffin, cut into 4 μm thick sections and then stained for MUC5AC following routine staining procedures. Sections were incubated overnight at 4°C with MUC5AC antibody (mouse monoclonal, 1:200, MA5-12178, Thermo Fisher Scientific, Waltham, MA, USA). Results were expressed as the intensity of staining and the percentage of IHC-positive cells to total epithelial area. Sections were scored 0, 1, 2, 3 points for colorless, light yellow, brown yellow and brown, respectively. Sections were them scored 0, 1, 2, 3, 4 points for 0, 1–25%, 26–50%, 51–75%, 76–100% of IHC-positive cells to total epithelial area, respectively. The final score was defined as the sum of the previous two scores. Each section had five randomly selected regions evaluated, after which the scores of the five regions were then averaged.

### Culture of Primary Human Nasal Epithelium Cells (PHNECs)

The cell culture was performed according to methods already published (11). Nasal polyps from the CRSwNP group were washed thoroughly and incubated in type XIV protease (p5147, Sigma, Burlington, MO, USA) in Dulbecco's Modified Eagle's Medium (DMEM, Thermo Scientific Inc., New York, USA) overnight at 4°C. The protease was neutralized with 5% bovine serum, and the epithelial cells were released from the tissues through vigorous shaking. After centrifugation and resuspension, the cells were plated on a plastic dish at 37°C for 1 h to eliminate fibroblasts. The cells were then cultured in bronchial epithelial growth medium (BEGM) at a density of 5 ×10^5^ cells/cm^2^ at 37°C in an atmosphere of 5% CO_2_ and 95% relative humidity.

### Real-Time Quantitative PCR

Total RNAs were isolated by phenol and guanidine isothiocyanate method using RNAiso Plus according to the manufacturer's instructions (TaKaRa, Kusatsu, Shiga, Japan). For amplification, MUC5AC sense (5′-GGA ACT GTG GGG ACA GCT CTT-3′), anti-sense (5′-GTC ACA TTC CTC AGC GAG GTC-3′), β2 microglobulin (β2M) sense (5′-GGA ACT GTG GGG ACA GCT CTT-3′) and anti-sense (5′-GTC ACA TTC CTC AGC GAG GTC-3′) primers were used. PCR was performed using the following mixture: 1 μl cDNA, 0.2 μl of each PCR primer, 0.2 μl ROX Reference DyeII (TaKaRa), 5 μl 5 × SYBR Premix Ex Taq (TaKaRa), 3 μl deionized water. Amplification of cDNA was performed using 45 cycles at 95°C 30 s, 95°C 15 s, 60°C 30 s and 72°C 1min. Data were normalized to the expression of GAPDH.

### Confocal Microscopy

PHNECs were plated on 6-well cell culture plates and allowed to adhere overnight, and then treated with and without recombinant human IL-19 (rhIL-19) (300 ng/ml, 1035-IL-025, R&D System, Minneapolis, MN,USA) for 24 h. All plates were washed three times with PBS and fixed for 10 min in 4% paraformaldehyde. Cells were washed three times with PBS and blocked for 30 min in 5% normal goat serum, and then incubated by MUC5AC antibody (mouse monoclonal, 1:50, MA5-12178, Thermo Fisher Scientific) at 4°C overnight. After washing with PBS, cells were incubated with Cy3-labeled goat anti-mouse IgG antibody (1:400, BS10006, Bioworld, St. Louis Park, MN, USA) for 1 h and DAPI (300 nm) for 5 min at room temperature. Immunofluorescence staining was imaged using confocal microscope at ×630 magnification.

### siRNA Transfection and STAT3 Inhibition

Human IL-20R2 siRNA (5′-GAUGGCUUCCACCUGGUUATT-3′) was purchased from TaKaTa. Briefly, a total of 5 ×10^5^ cells were plated in 24-well plates in triplicate and grown to ~30–50% confluency. Human IL-20R2 siRNA was transfected for 48 h using Lipofectamine™ 3000 Reagent (L3000015, Thermo Fisher Scientific) according to the protocol. Cells were then incubated with 300 ng/ml rhIL-19 or without for 24 h. Ten micrometer cryptotanshinone (CRY, HY-N0174, MedChemExpress, Monmouth Junction, NJ, USA), the inhibitor of STAT3 was added 1 h before rhIL-19 stimulation. The supernatants and cells were harvested for further analyses. Control groups without the addition of transfection reagents were also analyzed. All experiments were performed in triplicate.

### Western Blot

Total proteins were extracted from PHNECs and electrophoresis and transmembrane steps were run, respectively. The blotted membranes were incubated overnight at 4^0^C with antibodies targeting MUC5AC (1:100, ab24070, Abcam, Cambridge, MA, USA), STAT3 (1:2000, 12640, Cell Signaling Technology, Danvers, MA, USA), P-STAT3 (1:1000, 9131, Cell Signaling Technology) and GAPDH (1:3000, 10494-1-AP, Proteintech Group, Rosemont, IL, USA), and then developed using the ECL reagents. The relative intensity of each band, respectively, was normalized to GAPDH.

### MUC5AC ELISA

Supernatants were collected and subjected to ELISA. All protocols were carried out according to the manufacturer's instructions (E01M0350, BlueGene, Pudong New District, Shanghai, China).

### Statistical analyses

Statistics were performed using SPSS 20.0 software. All data are expressed as Means ±SEM. All data consisted of three or more independent tests or experiments. The Student *t*-test was performed to calculate differences between two groups. One-way ANOVA was used to determine statistically significant differences between three groups. *P*-values < 0.05 were considered significant.

## Results

### The Increased Expressions of Mucin and MUC5AC in Mucosa From Patients With CRS

**Figure 1:**
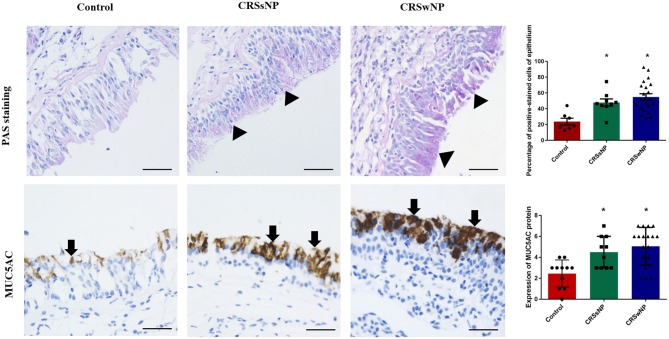
The expression of mucin and MUC5AC in the nasal tissues of the CRSsNP and CRSwNP patients and the normal controls. PAS staining shows that mucin is highly expressed in the epithelial cells of CRS patients (arrowhead); IHC staining shows that MUC5AC is highly expressed in the epithelial cells of CRS patients (arrows). Both mucin and MUC5AC are mildly expressed in the normal controls. No significant difference in mucin or MUC5AC immunoreactivity was observed between the CRSsNP and CRSwNP patients. Statistical significance was calculated according to the one-way ANOVA. ^*^P < 0.05 compared with the control group. Scale bars: 50 μm.

Although PAS staining demonstrated that mucin was more highly expressed in the epithelia and submucosal glands of the CRSwNP and CRSsNP patients in comparison with mucosa isolated from the control subjects without CRS, respectively (*P* < 0.05), the expression levels were not statistically significant between the groups (Figure 1). Mucin was mainly expressed on the apical side of the mucosal epithelia and submucosal glands.

Immunohistochemical staining showed strong staining of MUC5AC proteins in the mucosa tissues in patients with CRS, irrespective of nasal polyp presence, compared with the control group (Figure 1). MUC5AC was mainly expressed on the apical side of the mucosa epithelia. Since MUC5AC is a marker of goblet cell metaplasia, these results suggest metaplasia and hyperplasia of the nasal epithelial cells in CRS, resulting in MUC5AC over-production.

### The Co-expressions of IL-19 and MUC5AC in CRS

**Figure 2:**
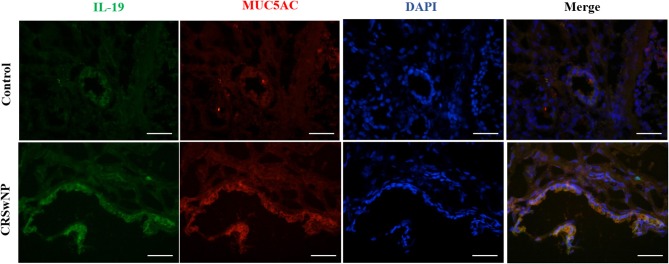
The co-expression of IL-19 and MUC5AC in the nasal tissues from the control and CRSwNP patients. Immunohistochemistry (Frozen Sections) shows that IL-19 (green) and MUC5AC (red) are highly expressed in the epithelial cells of control and CRSwNP patients. Nuclei are stained with DAPI (blue). Scale bars: 50 μm.

Since we have previously found that IL-19 and its receptors, IL-20R1 and IL-20R2, were more highly expressed in CRSwNP (Supplementary Figure 3), immunofluorescence was performed next to assess the relationship between IL-19 and MUC5AC. IL-19 was found to be co-expressed with MUC5AC in CRS mucosa (Figure 2). Both biomarkers were mainly co-localized in the epithelial cells in CRS tissues. These results suggest functional relevance of IL-19 to MUC5AC in mucosa tissue of patients with CRS.

### IL-19 Promoted the Expressions of MUC5AC

**Figure 3:**
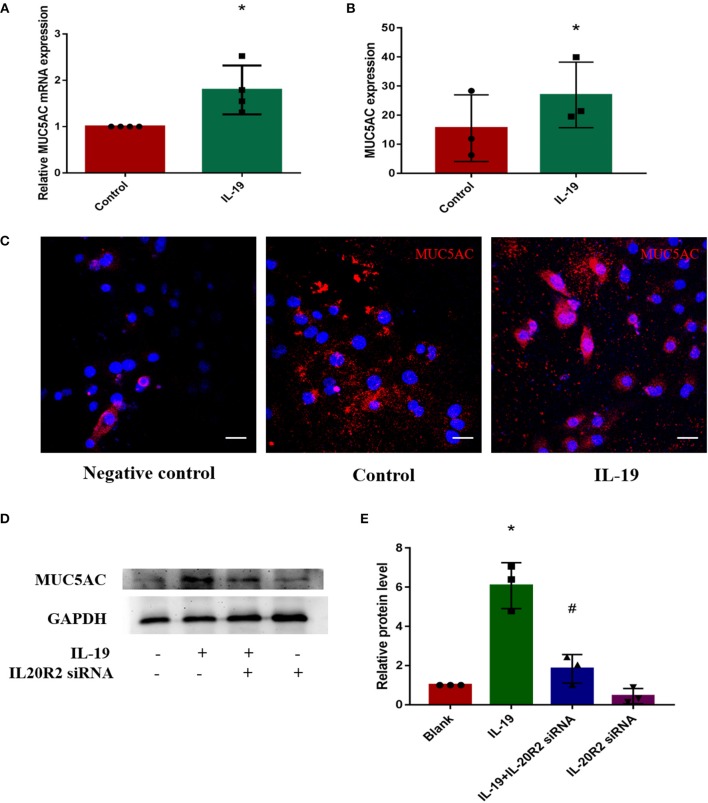
IL-19 up-regulated MUC5AC mRNA and protein expression in PHNECs. PHNECs were incubated with or without rhIL-19 (300 ng/ml) for 24 h. Real-time quantitative PCR was performed to measure the expression of MUC5AC transcript levels **(A)**. ELISA **(B)** and immunofluorescence **(C)** were performed to measure the expression of MUC5AC protein levels. Knockdown of IL-20R2 reduced MUC5AC expression induced by rhIL-19 in primary human nasal epithelial cells **(D,E)**. Western blotting was performed to measure the expression of MUC5AC protein levels. Statistical significance was calculated according to the Student *t*-test. ^*^P < 0.05 compared with the control group. ^#^*P* < 0.05 compared with the rhIL-19-treated group. Scale bars: 20 μm.

In order to determine whether IL-19 could promote MUC5AC expression in CRS, PHNECs were separated and cultured, then pretreated with rhIL-19. The optimal concentration of rhIL-19 is 300 ng/ml (Supplementary Figure 1). Real-time quantitative PCR was performed to measure mRNA while ELISA and confocal microscopy were performed to measure protein concentrations. Both mRNA and protein levels of MUC5AC were significantly higher in PHNECs incubated with rhIL-19 300 ng/ml for 24 h than in those incubated without rhIL-19 (Figures 3A–C), suggesting that IL-19 can up-regulate MUC5AC expression. To further determine specificity, we transfected the siRNA of IL-20R2, a receptor of IL-19, into PHNECs to interfere with IL-19 activity. As expected, MUC5AC expression was significantly decreased in siRNA transfected cells (Figures 3D,E). This observation indicates that IL-19 may promote goblet cell metaplasia, leading to the overproduction of MUC5AC and tissue remodeling in CRS.

### IL-19 Up-Regulated MUC5AC Production *via* STAT3 Pathway

**Figure 4:**
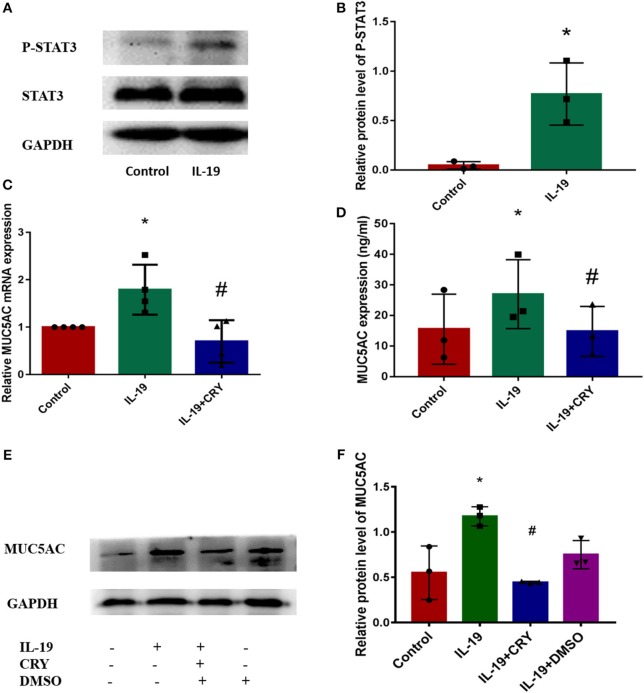
IL-19 up-regulated MUC5AC production in PHNECs *via* STAT3 pathway. rhIL-19 activated STAT3 pathway in PHNECs **(A,B)**. PHNECs were incubated with/out 300 ng/ml rhIL-19 for 24 h. Western blotting was performed to analyze the expression of P-STAT3 and STAT3 protein levels. The mRNA and protein expression of MUC5AC in PHNECs was decreased by the STAT3 inhibitor CRY. PHNECs were incubated with/out rhIL-19 (300 ng/ml) +CRY (10 μm) for 24 h. Real-time quantitative PCR was performed to measure the expression of MUC5AC transcript levels **(C)**. ELISA **(D)** and Western Blot **(E,F)** was performed to measure the expression of MUC5AC protein levels. Statistical significance was calculated according to the Student *t*-test or one-way ANOVA. ^*^P < 0.05 compared with the control group. ^#^*P* < 0.05 compared with the rhIL-19-treated group.

We next examined how IL-19 up-regulates MUC5AC. Similar to IL-6 (12–14), IL-19 binds its receptors and then activates STAT3 (15). Western Blot was performed to measure the expression and phosphorylation levels of STAT3 proteins in PHNECs after being pretreated by rhIL-19. The elevation of the phosphorylation levels of STAT3 was observed in PHNECs in rhIL-19 treated cells (Figures 4A,B). In order to determine whether IL-19 upregulated MUC5AC *via* activating STAT3, PHNECs were pretreated with CRY. As expected, the production of MUC5AC induced by rhIL-19 was suppressed significantly by CRY addition (Figures 4C–F), suggesting that IL-19 could activate the STAT3 pathway to promote MUC5AC expression.

## Discussion

Mucus is a critical component of the respiratory airway innate immune system, produced by goblet cells and submucosal glands, and normally coats the epithelial surfaces of the respiratory airways (2). Normal mucus secretion provides a beneficial barrier on airway surfaces both by retaining moisture and inhibiting noxious stimuli from the airway epithelium by normal ciliary clearance (16–18). However, in chronic respiratory inflammatory diseases, airway tissue remodeling occurs and the number of goblet cells increase, resulting in mucus over-production and airway obstruction (3, 18). Excessive mucus, therefore, disrupts ciliary clearance and obstructs airways, leading to the morbidity and mortality seen in chronic respiratory inflammatory diseases (19, 20). As a representative disease of chronic airway inflammation, CRS is also characterized by mucus over-production, manifesting as nasal discharge.

The major constituents of mucus are mucins (3), encoded by genes called MUCs. Up to now, although more than 20 mucin genes have been identified, MUC5AC is the most predominant mucin expressed in nasal epithelial cells. In murine airways, MUC5AC is regarded as a biomarker of goblet cell metaplasia (21, 22). It plays a vital role in the metaplasia of goblet cells after antigen challenge (23). Therefore, our study focuses on MUC5AC and hypothesizes that it is a target of mucus over-production in CRS.

In the present study, although we demonstrated that mucin was more highly expressed in the epithelia and submucosal glands of CRS patients in comparison with normal tissues, the expression levels were not significantly different between CRSwNP and CRSsNP. Concomitantly, immunohistochemical staining showed that MUC5AC proteins were strongly stained in CRS patients, irrespective of nasal polyp presence, compared with the control group. This resemblance between CRSwNP and CRSsNP patients can be attributed to the effects of chronic inflammation, ciliary impairment, and tissue edema. CRSwNP and CRSsNP patients are known to undergo long-term inflammatory challenges, tissue remodeling and increased mucus production (4), responsible for their clinical manifestations.

We report the precise location of MUC5AC to be mainly expressed on the apical side of the mucosal epithelia of CRS patients associated with nasal epithelial cell metaplasia and hyperplasia, appearing with chronic inflammation stimulation and resulting in MUC5AC over-production, as previously reported (7, 8, 23).

Recent studies have shown that the IL-20 subfamily participates in tissue remodeling and plays an important role in mucin deposition on epithelial cells in inflammatory bowel disease (10). IL-19, a crucial member of the IL-20 subfamily, is associated with several inflammatory diseases and autoimmune diseases, participating in the Th2 type immunological response (24–26). It was recognized to be highly expressed in airway epithelia of asthmatic patients (27). In our previous study, we presented that IL-19 and its receptors, IL-20R1 and IL-20R2, were more expressed in CRSwNP (Supplementary Figure 3). Therefore, we suppose that IL-19 is involved in mucus production in the epithelial cells in CRS.

In the present study, we observed that the expressions of IL-19 and MUC5AC increased in CRS mucosa. Both of them were mainly expressed in the epithelial cells in CRS tissues. In order to determine whether IL-19 contributes to the increased MUC5AC expression in CRS, we separated and cultured the PHNECs and confirmed that rhIL-19 stimulated the MUC5AC expression while this promotion was eliminated by IL-19 receptor knock-down using siRNA transfection method. To the best of our knowledge, this is first study showing that IL-19 is involved in the pathogenesis of patients with CRS. As this is a human study, future studies on the specific role of IL-19 in the production of MUC5AC and pathogenesis of CRS in animal models, using IL-19 knock-out or IL-19 transgenic mice, is warranted.

Several signaling pathways have been reported to be involved in mucus overproduction. However, little information is available on the exact mechanism of mucus regulation. To date, the most frequently reported pathways include STAT6 (23, 28–30), ERK1/2 (31, 32) and NF–κB (33–35). STAT proteins are critical mediators of cytokine signaling among which STAT3 has been identified to be linked to tumorigenesis and inflammation (12, 36–38). Previous studies proposed that STAT3 and its phosphorylated form, P-STAT3, were mainly located in the superficial layer of the epithelium of CRSwNP patients, while hardly expressed in normal tissues (39, 40), similar to the expressions of IL-19 and MUC5AC observed in the present study. We wonder if the effect of STAT3 in IL-19, induced MUC5AC production. Recent studies have connected STAT3 with the production and secretion of MUC5AC (41–43). Furthermore, the STAT3 pathway was activated by IL-19 and its subfamily members in the keratinocyte cell line (15) and human keratinocytes in previous observations (44–46). In the present study, we provide a new line of evidence that IL-19 activates the STAT3 pathway in airway epithelial cells. Our results also further validate our proposal that IL-19 up-regulates MUC5AC production in chronic rhinosinusitis *via* the STAT3 pathway. CRY, the main active component of saliva, was supposed to inhibit the STAT3 pathway (47). When PHNECs were pretreated with CRY and IL-19, the gene and protein expressions of MUC5AC were suppressed. Although the role of other signal pathways cannot be excluded, our study proposes that STAT3 is mainly involved in MUC5AC gene and protein expressions induced by IL-19 in CRS.

In summary, we propose that IL-19 up-regulates MUC5AC production *via* the STAT3 pathway. This is the first demonstration that IL-19 is involved in mucus production in airway epithelium and determines the exact mechanism, suggesting that IL-19 plays an important role in mucin production in chronic respiratory diseases and target IL-19 and its downstream signal pathway could have a therapeutic potential in patients with CRS.

## Data Availability

All datasets generated for this study are included in the manuscript/Supplementary Files.

## Ethics Statement

This study was carried out in accordance with the recommendations of the ethics committee for clinical medical research at the Third Affiliated Hospital of Sun Yat-sen University (No.[2016]2-26). All enrolled subjects provided written informed consent.

## Author Contributions

GZ and SZ designed the research and was in charge of correspondence. XLa, XLi, and LC performed the experiments. XC, ZH, HB, and JH helped with carrying out the experiments. XLa, LY, XW, and ZW analyzed the data. XLa, LC, GZ, and SZ wrote the manuscript. JB revised the manuscript. All the authors read and approved the final manuscript.

### Conflict of Interest Statement

The authors declare that the research was conducted in the absence of any commercial or financial relationships that could be construed as a potential conflict of interest.
