# Supplementary material for: IL-19 Up-Regulates Mucin 5AC Production in Patients With Chronic Rhinosinusitis via STAT3 Pathway
Source: Front Immunol. 2019 Jul 17;10:1682. doi: 10.3389/fimmu.2019.01682 (PMC6660249; doi:10.3389/fimmu.2019.01682)
Supplement: Supplementary file 1 [file Data_Sheet_1.pdf]

Supplementary Material

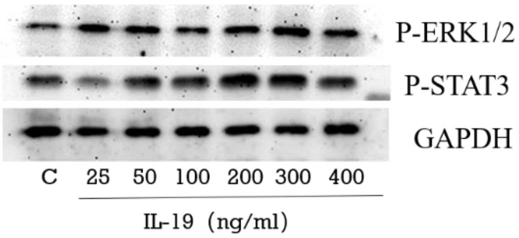

Supplementary Figure 1. rhIL-19 activated STAT3 and ERK1/2 pathways in PHNECs. PHNECs were treated with various concentrations of rhIL-19 (0, 25, 50, 100, 200, 300 ng/mL) for 24 h. Western blot was performed to analyze the expression of P-STAT3 and P-ERK1/2 protein levels. Results are representative of three independent experiments.

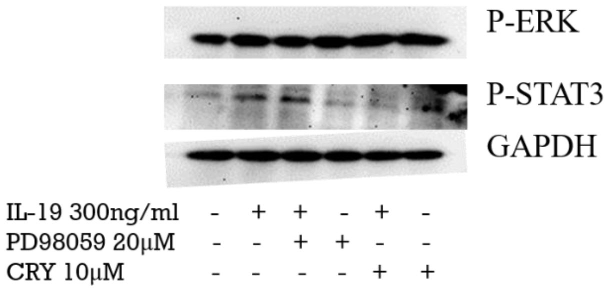

Supplementary Figure 2. The expression of P-STAT3 and P-ERK1/2 protein levels in PHNECs. PHNECs were pretreated with CRY (an inhibitor of STAT3) and PD98059 (an inhibitor of ERK1/2) before rhIL-19 stimulation. Western blotting was performed to analyze the expression of P-STAT3 and P-ERK1/2 protein levels. Results are representative of three independent experiments.

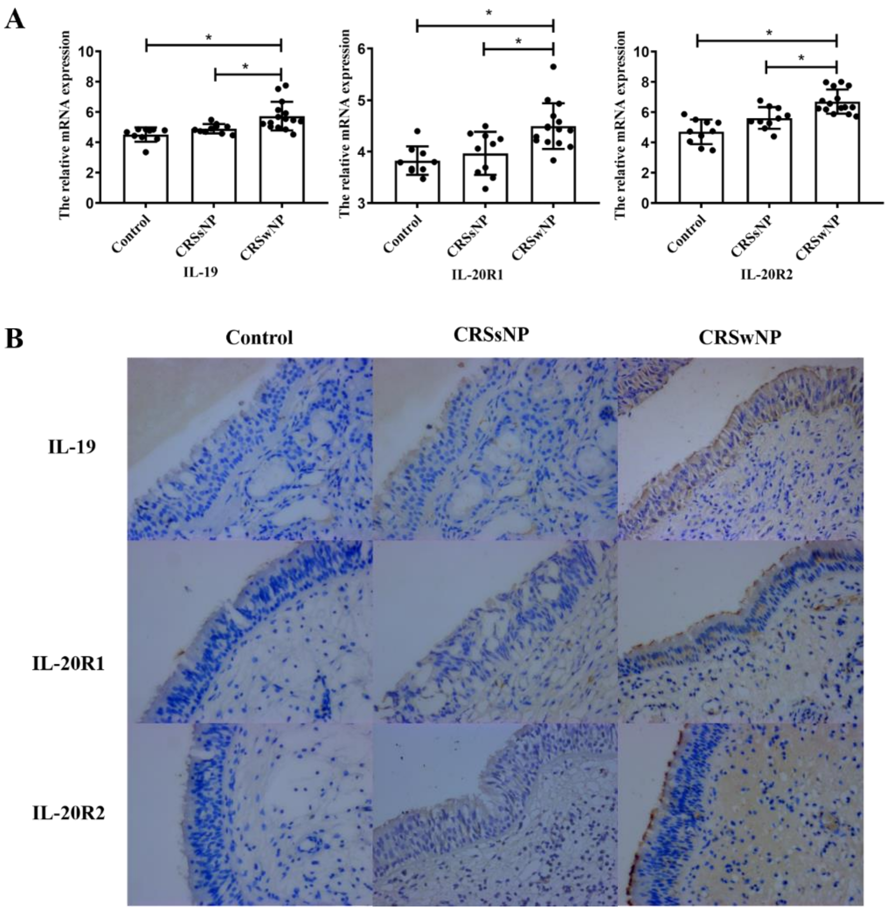

Supplementary Figure 3. The expression of IL-19 and its receptors in the nasal tissues from the CRSsNP and CRSwNP patients and the normal controls. Real-time quantitative PCR was performed to measure the gene expressions of IL-19 and its receptors, IL-20R1 and IL-20R2, in grinding tissues from the CRSsNP (n=10) and CRSwNP (n=15) patients and the normal controls (n=10) (A). IHC staining shows that IL-19, IL-20R1 and IL-20R2 were higher expressed in CRSwNP and mainly expressed on the apical side of the mucosal epithelia (B). Statistical significance was calculated according to the one-way ANOVA. *P <0.05.
